# Exploiting TERT dependency as a therapeutic strategy for NRAS-mutant melanoma

**DOI:** 10.1038/s41388-018-0247-7

**Published:** 2018-04-26

**Authors:** Patricia Reyes-Uribe, Maria Paz Adrianzen-Ruesta, Zhong Deng, Ileabett Echevarria-Vargas, Ilgen Mender, Steven Saheb, Qin Liu, Dario C. Altieri, Maureen E. Murphy, Jerry W. Shay, Paul M. Lieberman, Jessie Villanueva

**Affiliations:** 1 Molecular & Cellular Oncogenesis Program, The Wistar Institute, Philadelphia, PA USA; 2 Gene Expression & Regulation Program, The Wistar Institute, Philadelphia, PA USA; 3 Department of Cell Biology, University of Texas Southwestern Medical Center, Dallas, TX USA; 4 Immunology, Microenvironment & Metastasis Program, The Wistar Institute, Philadelphia, PA USA; 5 Melanoma Research Center, The Wistar Institute, Philadelphia, PA USA

## Abstract

Targeting RAS is one of the greatest challenges in cancer therapy. Oncogenic mutations in *NRAS* are present in over 25% of melanomas and patients whose tumors harbor NRAS mutations have limited therapeutic options and poor prognosis. Thus far, there are no clinical agents available to effectively target NRAS or any other RAS oncogene. An alternative approach is to identify and target critical tumor vulnerabilities or non-oncogene addictions that are essential for tumor survival. We investigated the consequences of NRAS blockade in NRAS-mutant melanoma and show that decreased expression of the telomerase catalytic subunit, TERT, is a major consequence. TERT silencing or treatment of NRAS-mutant melanoma with the telomerase-dependent telomere uncapping agent, 6-thio-2′-deoxyguanosine (6-thio-dG), led to rapid cell death, along with evidence of both telomeric and non-telomeric DNA damage, increased ROS levels, and upregulation of a mitochondrial antioxidant adaptive response. Combining 6-thio-dG with the mitochondrial inhibitor Gamitrinib attenuated this adaptive response and more effectively suppressed NRAS-mutant melanoma. Our study uncovers a robust dependency of NRAS-mutant melanoma on TERT, and provides proof-of-principle for a new combination strategy to combat this class of tumors, which could be expanded to other tumor types.

## Introduction

Significant improvement in the treatment of melanoma has been achieved through the use of targeted- and immuno-therapies [1]. Despite this progress, a large percentage of patients do not benefit from these therapies and/or experience disease progression. In particular, melanomas with NRAS mutations are highly resistant to most therapies and have poor prognosis [2–4].

NRAS is the second most frequently mutated oncogene in melanoma [5, 6]. In addition to mutations in NRAS, mutations in NF1 (>10%), or activation of receptor tyrosine kinases (RTKs), can also activate RAS signaling in melanoma [7–9]. Furthermore, a frequent mechanism of acquired resistance to BRAF/MEK inhibitors is mediated by secondary mutations in NRAS [10, 11]. Consequently, ~40% of melanoma patients have tumors that are driven by aberrant NRAS signaling. Targeting RAS has been remarkably challenging; thus far, there are no drugs in the clinic that directly target mutant NRAS. Alternative approaches, including the use of antagonists of RAS effectors, including RAF and PI3K, have had limited success for the treatment of NRAS-driven metastatic melanoma [2, 12]. Therefore, there is an urgent need to identify vulnerabilities in this tumor type that can be exploited therapeutically.

TERT, the catalytic subunit of telomerase, is a promising therapeutic target for cancer, as it is highly expressed in most tumor cells and seldom expressed in most normal cells [13, 14]. Mutations in the TERT promoter have been identified in >70% of melanomas, constituting the most frequent genetic alteration in these tumors [5, 15, 16]. These mutations create de novo Ets/TCF (E-twenty six/ternary complex factor) binding sites, enhancing the expression of TERT in these cells [5, 15]. Clinically, *BRAF* or *NRAS*-mutant melanoma patients whose tumors have *TERT* promoter mutations have poor overall survival compared to patients with tumors with a non-mutated *TERT* promoter [17]. These data suggest that TERT is a key player in melanoma and a compelling therapeutic target. In addition to its canonical role in maintaining telomere length, TERT has been recognized to regulate extra-telomeric processes [18–22]. For example, TERT has been shown to regulate apoptosis, DNA damage responses, chromatin state, and cellular proliferation [23–28]. These combined data suggest that TERT-based strategies might have valuable therapeutic effects.

Developing clinically relevant approaches to inhibit TERT has been daunting. Most TERT inhibitors evaluated thus far target the enzymatic activity of telomerase and rely on critical shortening of telomeres to kill tumor cells; consequently, there is a prolonged lag period for efficacy [29, 30]. This prolonged period could constitute a potential disadvantage, as cancer cells can rapidly adapt to the pharmacological challenges and become resistant. In addition, the long duration of treatment could lead to increased toxicity and/or decreased tolerability. Hence, novel TERT-based therapeutic strategies that can elicit relatively rapid and sustained effects could have significant impact on cancer treatment. Here, we hypothesized that resistance to TERT inhibition depends on the activation of an adaptive response, which can be exploited for drug combination strategies providing novel avenues to combat NRAS-driven melanoma.

## Results

### NRAS-mutant melanoma is addicted to TERT

**Fig. 1:**
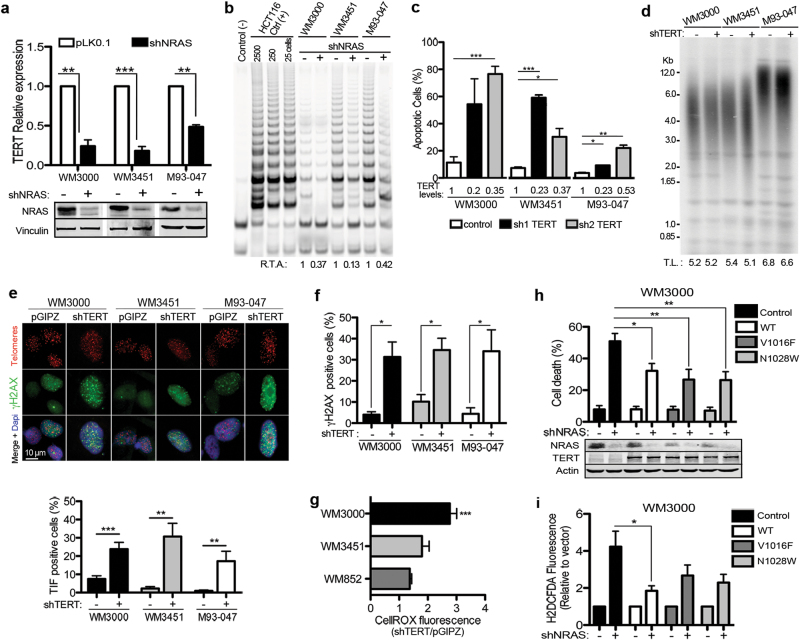
Depletion of TERT induces rapid death of NRAS-mutant melanoma cells. **a**, **b** NRAS^mut^ melanoma cells were transduced with lentiviruses enconding NRAS shRNA (+) or non-targeting empty vector control (−). **a** NRAS levels were assessed by immunoblotting (bottom panel) and TERT mRNA levels were assessed by qRT-PCR. **b** Relative telomerase activity (R.T.A.) was determined by TRAP assays following NRAS depletion in NRAS^mut^ melanoma cells. Negative control (−): primer; positive control (+): HCT116 cells. **c**–**g** TERT was depleted using two different hairpins (sh1, sh2). Apoptosis was determined by flow cytometry using the Annexin V analog PSVue in cells transduced with TERT shRNA 10 days post infection (dpi). TERT mRNA levels are shown below the corresponding bar as fold change relative to vector control (**c**). **d** Telomere length was assessed by Southern blotting of terminal restriction fragments (TRF) in NRAS^mut^ cells 10 days after transduction. (−): empty vector, pGIPZ; (+): TERT shRNA. Mean TRF length expressed as kilobase pairs is indicated at the bottom. **e**–**g** NRAS^mut^ melanoma cells were transduced with TERT shRNA. Telomere dysfunction-induced foci (TIF) was determined 7 dpi by indirect immunofluorescence combined with fluorescence in situ hybridization (FISH). Bottom panel, quantification of TIF assays from two independent experiments. Cells were scored as TIF positive when 4 or more γH2AX foci (green) co-localized with telomere foci (red) (**e**). DNA damage was assessed by γH2AX staining by flow cytometry (**f**). Reactive oxygen species (ROS) levels were measured by CellRox-Deep Red staining by FACS (**g**). **h**, **i** WM3000 cells were transduced with wild type (WT) or catalytically impaired VYLF1016 (V1016F), VYFL1028 (N1028W) TERT-mutant constructs, followed by shRNA-mediated NRAS silencing. Cell death was assessed by FACS measurement of Annexin V and propidium iodide (PI) staining (**h**). ROS levels were measured by H2DCFDA fluorescence (**i**). pLKO.1 and pBlast are empty vectors for NRAS shRNA and TERT constructs, respectively. Data represent average of three independent experiments ± SEM. *p < 0.05; **p < 0.01; ***p < 0.005 determined by unpaired Student’s *t*-test

To identify specific vulnerabilities of NRAS-mutant melanoma, we performed gene expression analysis in NRAS-mutant melanoma cells following depletion of NRAS. We focused on genes known to regulate proliferation and senescence, as we had established that NRAS silencing rapidly triggered proliferation arrest and induced senescence. One of the most pronounced effects of NRAS silencing was downregulation of the catalytic subunit of telomerase, TERT (Fig. 1a; Supplementary Figure 1). Of note, TERT levels were downregulated following NRAS depletion in both NRAS-mutant melanoma cells harboring TERT promoter mutations and to a lesser degree in melanoma cells with wild-type TERT promoter (Supplementary Table 1). Downregulation of TERT was coupled to diminished telomerase activity (60–90%; Fig. 1b) [31]. Consistent with previous reports indicating that the RAS/MEK signaling pathway regulates TERT expression [32, 33], treatment of NRAS-mutant melanoma cells with the MEK inhibitor trametinib downregulated TERT mRNA levels (Supplementary Figure 2). As telomerase activity is associated with cell cycle progression [34], we wondered whether the decrease in TERT levels could be a bystander effect of cell cycle arrest elicited by NRAS silencing. To rule out this possibility, we treated NRAS-mutant melanoma cells with a cdk4/6 inhibitor to induce cell cycle arrest and assessed TERT levels. Treatment with the cdk4/6 inhibitor palbociclib (PD-0332991) led to sustained proliferation arrest, but did not significantly affect TERT levels (Supplementary Figure 3).

To determine the dependency of NRAS-mutant melanomas on TERT, we evaluated the effect of TERT silencing in these cells (Fig. 1c). Depletion of TERT, using two different short hairpins, led to extensive and relatively rapid (7–10 days) induction of apoptosis in NRAS-mutant melanoma cells, with no evidence of senescence within this time frame (Fig. 1c; Supplementary Figure 4a–c). In addition, we did not detect a significant decrease in average telomere length within 10 days following transduction with shTERT (Fig. 1d), when much of cell death (~70%) occurred. TERT depletion led to increased telomere dysfunction-induced foci (TIF), increased phosphorylation of histone γ-H2AX, and accumulation of reactive oxygen species (Fig. 1e–g; Supplementary Figure 5). To determine whether oxidative stress was contributing to the telomere dysfunction, cells transduced with non-targeting vector or TERT shRNA were treated with the antioxidant *N*-acetyl-l-cysteine (NAC). Treatment with NAC attenuated the number of TIF in TERT-depleted cells, suggesting that oxidative stress contributes to the telomeric DNA damage and dysfunction (Supplementary Figure 6). Together, these data support the premise that TERT depletion may lead to both telomeric and non-telomeric DNA damage in NRAS-mutant melanoma cells.

We next sought to clarify the requirement for the catalytic activity of TERT for the pro-survival function of this protein in NRAS-mutant melanoma. Toward this end, we compared the ability of ectopically expressed wild-type TERT (WT; catalytically active) with two different catalytic domain-deficient constructs of TERT for their ability to rescue cell death induced by NRAS silencing. These two mutants, FVYL1016 and FVYL1028, contain point mutations in the FVYL motif, which impair its catalytic activity and lead to telomere attrition in primary human fibroblasts [35] and melanoma cells ectopically expressing these constructs (Supplementary Figure 7a). Of note, mutations within this motif do not affect proper protein folding [35] or proliferation of NRAS-mutant melanoma cells ectopically expressing these constructs (Supplementary Figure 7b). Ectopic expression of wild-type (WT) TERT, or either of the two catalytically impaired TERT mutants, FVYL1016 or FVYL1028, partially protected melanoma cells from cell death induced by NRAS depletion (Fig. 1h). Similarly, ectopic expression of either WT-TERT, or to a lesser extent either of the two TERT mutants could alleviate the increased levels of ROS associated with NRAS silencing (Fig. 1i). These combined data indicate that TERT contributes to the survival of NRAS-mutant melanoma and raises the possibility that, in addition to its catalytic role, TERT might also possess a non-catalytic survival role in melanoma.

### Telomere dysfunction is coupled to induction of an antioxidant adaptive response program

**Fig. 2:**
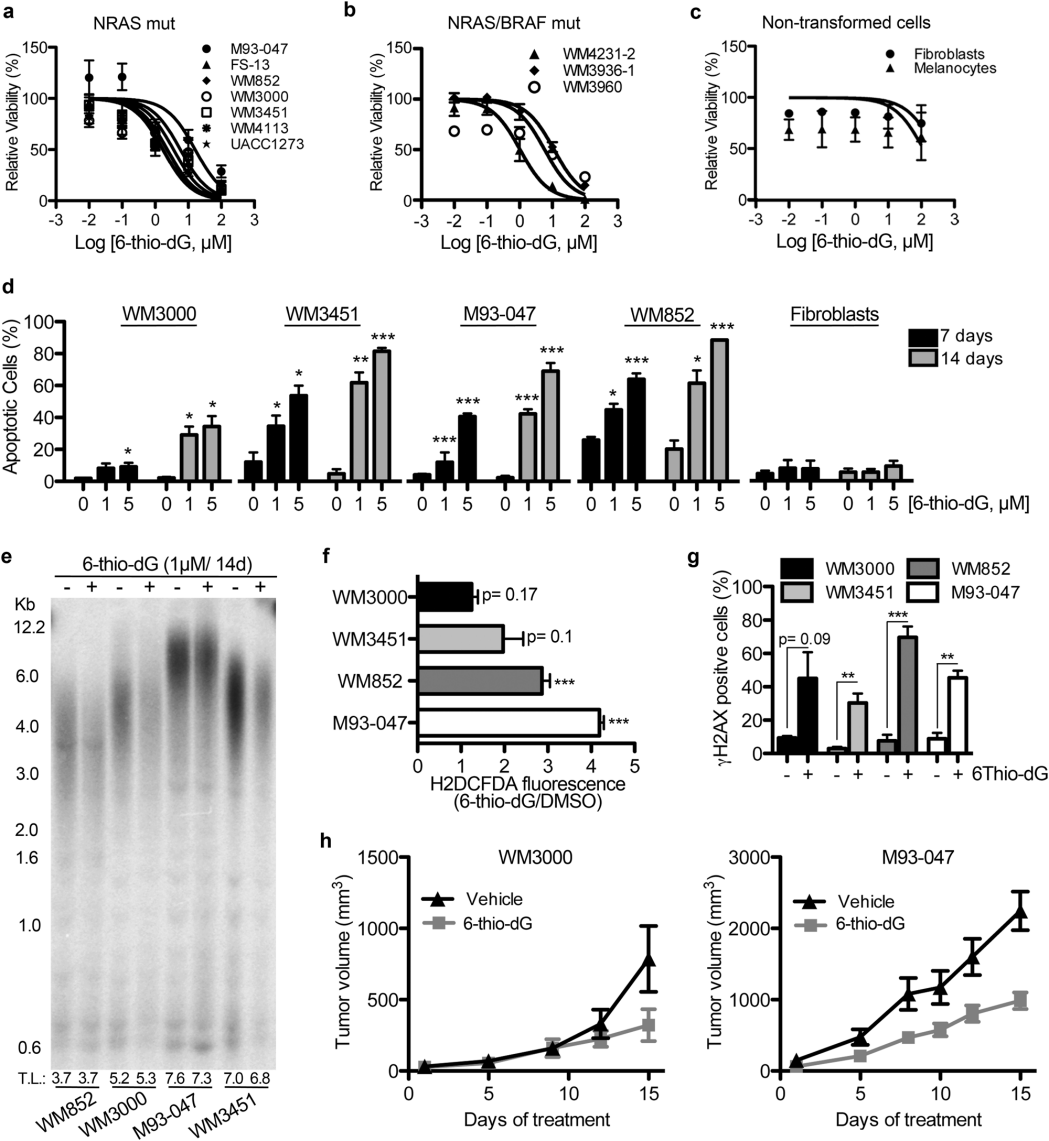
6-thio-dG impairs viability of NRAS-mutant melanoma. **a**–**c** NRAS^mut^ melanoma cells (**a**), BRAF-V600E/NRAS-mutant cells derived from melanoma patients resistant to BRAF/MEK inhibitors (**b**), or non-transformed melanocytes and fibroblasts (**c**) were treated with 6-thio-dG for 7 days. Cell viability was measured by Alamar Blue assay and calculated relative to DMSO-treated cells. **d** NRAS^mut^ melanoma cells and non-transformed human fibroblasts were treated with 1 or 5 μM 6-thio-dG for 7 or 14 days. Cell death was assessed by PSVUe staining. Percent of apoptotic cells is shown. **e** Cells were treated with DMSO or 1 µM 6-thio-dG for 14 days and telomere length was assessed by Southern blotting of TRF. Mean TRF length expressed as kilobase pairs is indicated at the bottom. **f**, **g** Cells were treated with 1 µM 6-thio-dG for 7 days. **f** ROS production was measured by H2DCFDA fluorescence and normalized to DMSO-treated cells. **g** DNA damage was assessed by γH2AX staining. Data represent average from three independent experiments ± SEM. *p < 0.05; **p < 0.01; ***p < 0.005 in unpaired Student’s *t*-test. **h** Mice bearing established WM3000 or M93-047 NRAS^mut^ tumors were treated with vehicle control or 6-thio-dG (2.5 mg/kg i.p; q.d). Tumor volume was measured and average tumor volume (*n* = 4) ± SEM was plotted vs. time

To determine the therapeutic value of exploiting melanoma cell dependency on TERT, we used the nucleoside analog 6-thio-dG-2′-deoxyguanosie (6-thio-dG) [36]. 6-thio-dG is a telomerase substrate precursor that is rapidly incorporated into the telomeres of cells expressing telomerase, acting as an uncapping agent and leading to a rapid induction of TIFs [36]. 6-thio-dG has no significant effects on red blood cells, white blood cells, alanine aminotransferase (ALT), aspartate aminotransferase (AST), and creatinine levels in mice [8]. Treatment with 6-thio-dG impaired the viability of NRAS-mutant melanoma cells (Fig. 2a, b; Supplementary Figure S8a–b), including cells driven by the secondary mutations in NRAS derived from melanoma patients with acquired resistance to BRAF and MEK inhibitors (Fig. 2b). As expected, 6-thio-dG did not significantly affect telomerase-negative cells such as non-transformed primary human melanocytes or fibroblasts (Fig. 2c). Treatment of NRAS-mutant melanoma cells with 6-thio-dG led to cell death after 7–14 days (Fig. 2d), without evidence for significant telomere shortening after 14 days (Fig. 2e). Similar to the effects of TERT depletion, treatment with 6-thio-dG led to increased levels of ROS (Fig. 2f), TIFs (Supplementary Figure 8c, d), and γH2AX (Fig. 2g), thus phenocopying the effects of TERT silencing. Further, treatment of NRAS-mutant tumor-bearing mice with 6-thio-dG slowed the growth of tumors (Fig. 2h).

**Fig. 3:**
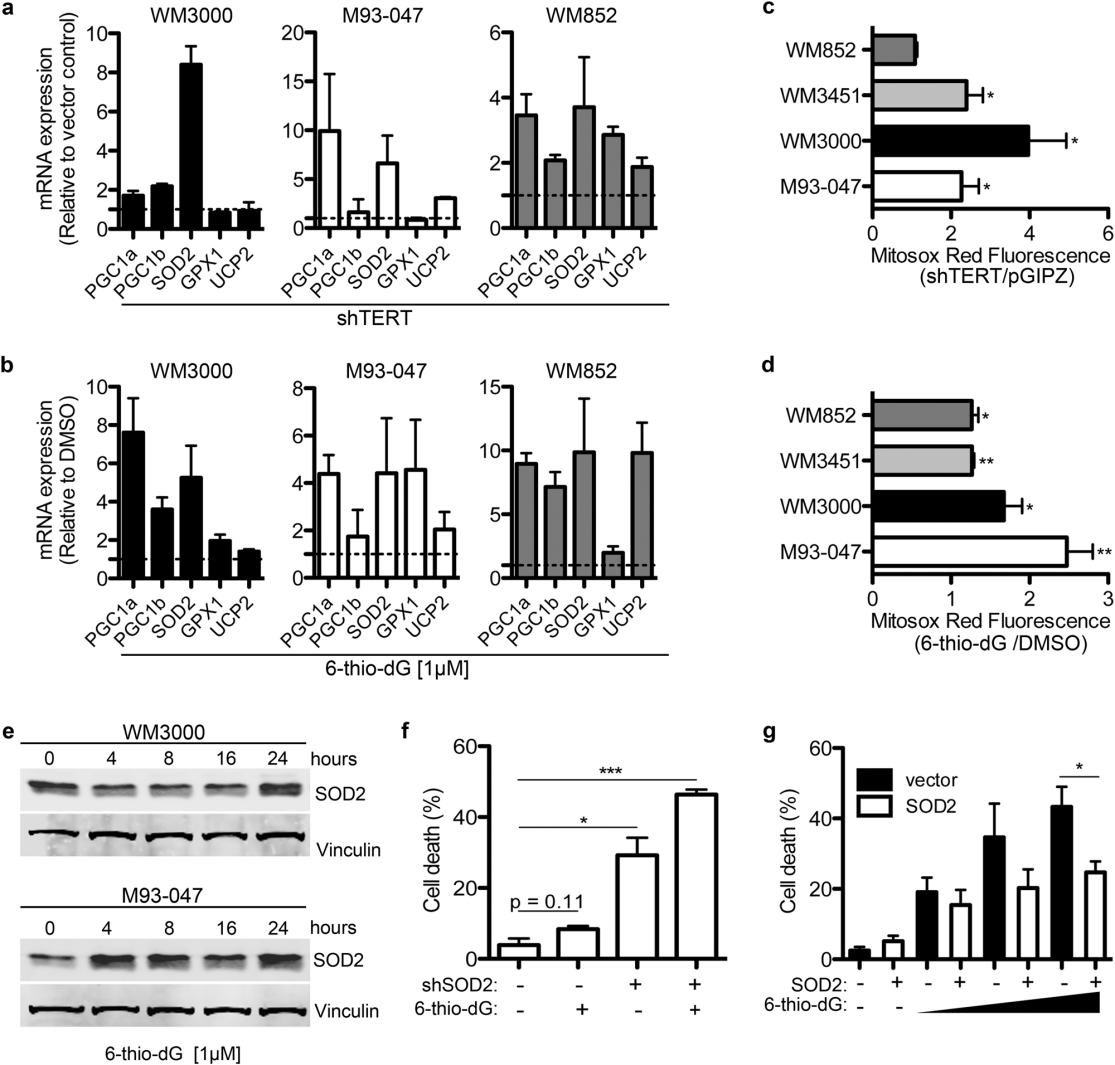
Telomere dysfunction enhances oxidative stress leading to upregulation of an antioxidant program. **a**–**d** NRAS^mut^ melanoma cells were transduced with TERT shRNA (**a**, **c**) or treated with 1 µM 6-thio-dG for 7 days (**b**, **d**). mRNA levels of the indicated genes were quantified by qRT-PCR (**a**, **b**). Mitochondrial superoxide production was measured by MitoSox Red fluorescence (**c**, **d**) and expressed as fluorescence relative to vector or DMSO controls. **e** NRAS^mut^ melanoma cells were treated with 1 µM of 6-thio-dG for the indicated times and SOD2 protein levels measured by Western blotting. **f** SOD2 was silenced using shRNA. Transduced M93-047 cells were treated with DMSO or 1 µM of 6-thio-dG and cell death was assessed by Annexin V/PI straining. **g** M93-047 cells were transduced with a SOD2 lentiviral construct or empty vector control (pLX304). Transduced cells were treated with increasing doses (1, 2.5, and 5 µM) of 6-thio-dG for 7 days and cell death was assessed. Data represent average from three independent experiments ± SEM. *p < 0.05; **p < 0.01; ***p < 0.005 in unpaired Student’s *t*-test

We noted that TERT depletion or treatment with 6-thio-dG led to upregulation of several detoxifying enzymes such as SOD2, GPX1, and UCP2 (Fig. 3a, b), as well as other enzymes involved in mitochondrial function and redox balance. Consistently, TERT depletion or treatment with 6-thio-dG led to increased mitochondrial ROS levels (Fig. 3c, d). We next sought to determine whether the most consistently upregulated antioxidant enzyme, SOD2 (Fig. 3a,b,e), could be counteracting oxidative stress associated with telomere uncapping, and enabling tumor cells to survive with high levels of ROS. Depletion of SOD2 enhanced the ability of 6-thio-dG to induce apoptosis of NRAS-mutant melanoma cells (Fig. 3f; Supplementary Figure 9a). Conversely, overexpression of SOD2 attenuated the apoptosis-inducing effects of 6-thio-dG (Fig. 3g; Supplementary Figure 9b).

### Co-targeting the mitochondria potentiates the anti-melanoma effects of 6-thio-dG

**Fig. 4:**
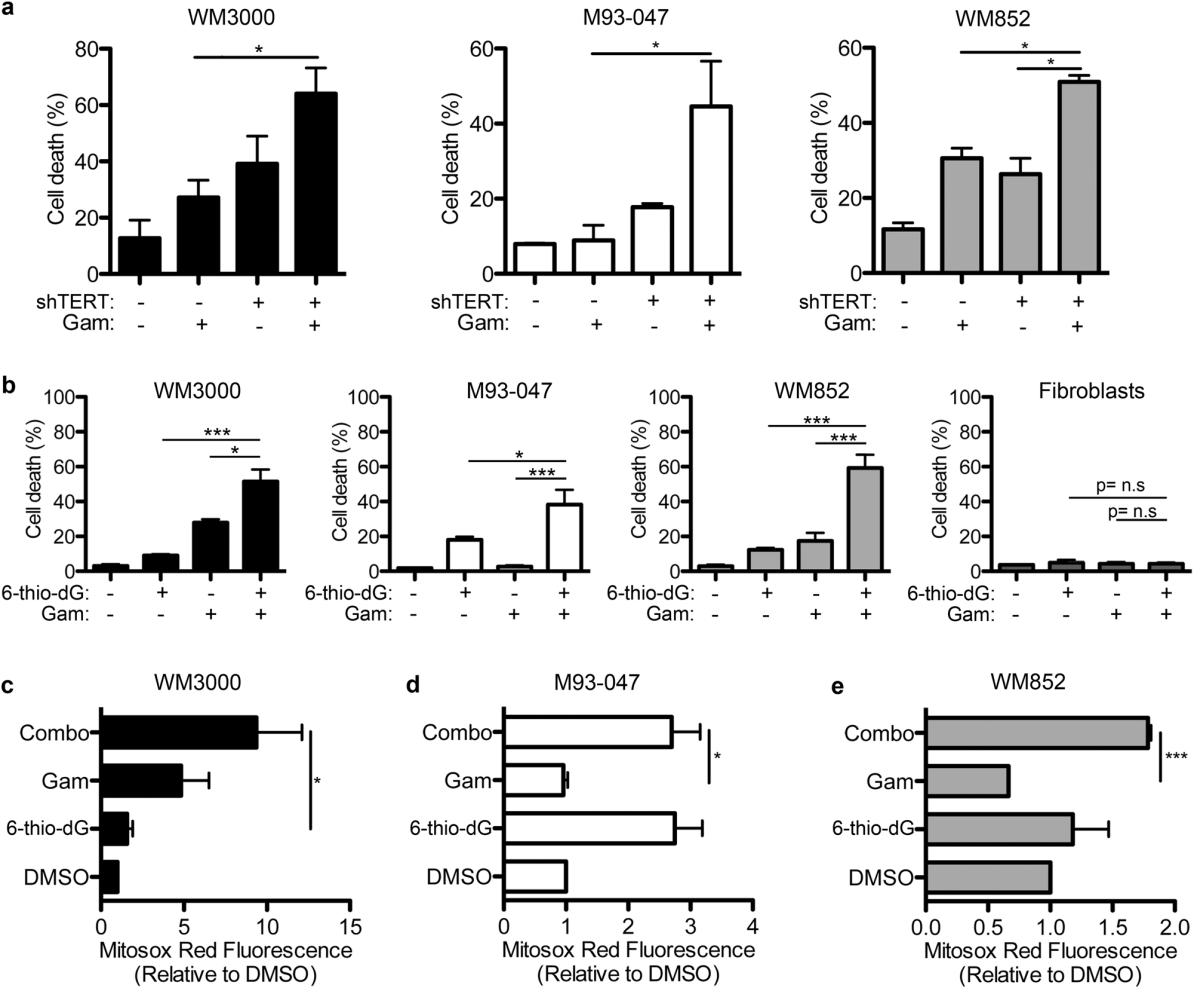
Concomitant induction of telomere and mitochondrial dysfunction triggers cell death in NRAS-mutant melanoma. **a** NRAS^mut^ melanoma cells transduced with TERT shRNA were treated 7 days post infection with 5 µM Gamitrinib (Gam) for 48 h. Cell death was assessed by Annexin V and PI staining. **b** NRAS^mut^ melanoma cells and non-transformed fibroblasts were treated with 1 μM 6-thio-dG for 5 days. At day 5, culture medium was replaced and cells treated with 1 μM 6-thio-dG plus 5 μM Gam for two more days. Cells were stained with Annexin V and PI and percent cell death was determined by flow cytometry. **c**–**e** Cells were treated as in **b** and mitochondrial ROS levels were measured using Mitosox Red by FACS. Data represent average of three independent experiments ± SEM. *p*-values were calculated using unpaired Student’s *t*-test; *p < 0.05; ***p < 0.005

On the basis of the results described above, we hypothesized that combining a mitochondria disrupting agent with 6-thio-dG or TERT depletion could kill NRAS-mutant melanoma cells by concurrently inducing high levels of ROS and blocking the antioxidant adaptive program trigerred by telomere dysfunction. We tested this hypothesis using Gamitrinib, an ATPase antagonist that disrupts mitochondrial function by selectively inhibiting mitochondrial Hsp90 [37]. Importantly, Gamitrinib has been shown to downregulate SOD2 levels [38]; accordingly, depletion of TERT enhanced the cytotoxic effects of Gamitrinib (Fig. 4a). In addition, pre-treatment of NRAS-mutant melanoma cells with 6-thio-dG-sensitized cells to the cytotoxic effects of Gamitrinib, enhancing cell death (Fig. 4b). This combination had minimal effects on non-transformed cells, such as human primary fibroblasts (Fig. 4b). In addition, 6-thio-dG in combination with Gamitrinib led to increased levels of mitochondrial ROS in NRAS-mutant melanoma cells (Fig. 4c–e).

**Fig. 5:**
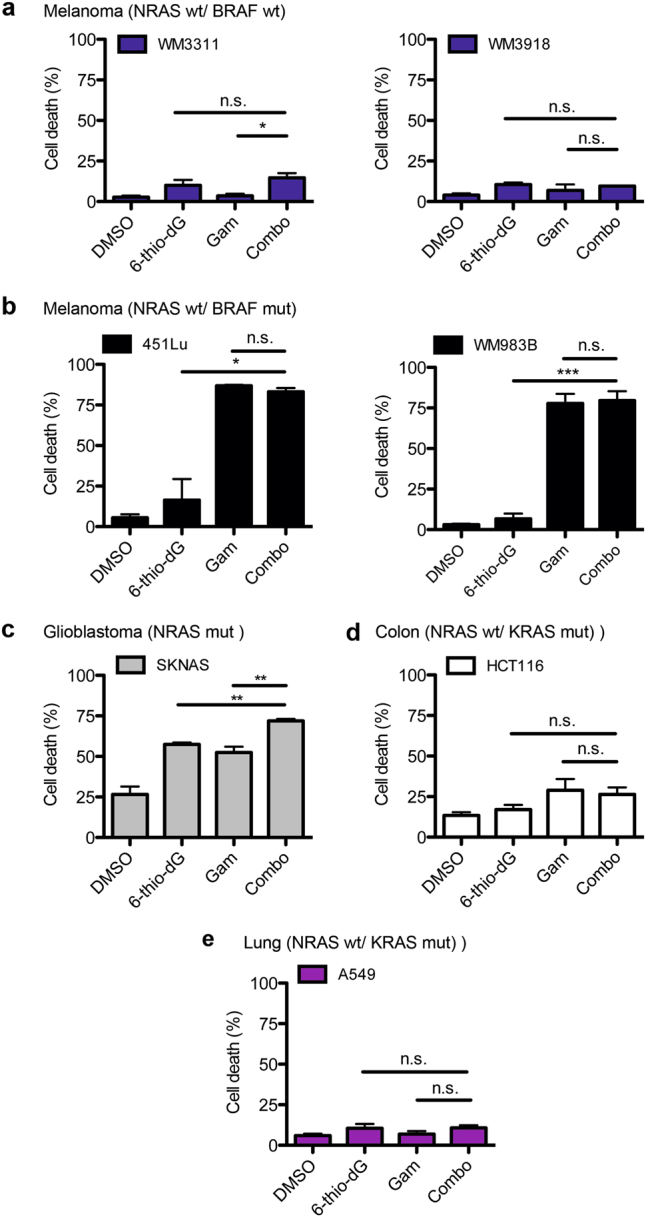
Combination treatment with 6-thio-dG and Gamitrinib triggers death selectively in NRAS-mutant tumor cells. **a**–**e** NRAS-WT/BRAF-WT melanoma cells (**a**), NRAS-WT/BRAF-mut melanoma cells (**b**), NRAS-mutant glioblastoma cells (**c**), NRAS-WT/KRAS-mutant colon cancer cells (**d**), and NRAS-WT/KRAS-mutant lung cancer cells (**e**) were treated with 1 μM 6-thio-dG for 5 days. At day 5, culture medium was replaced and cells treated with 1 μM 6-thio-dG plus 5 μM Gam for two more days. Cells were stained with Annexin V and PI and percent cell death was determined by FACS. Data represent average of three independent experiments ± SEM; *p*-values were calculated using unpaired Student’s *t*-test; *p < 0.05; **p < 0.001

To determine whether Gamitrinib could potentiate the efficacy of 6-thio-dG in melanoma cells lacking NRAS mutations, we treated NRAS-WT melanoma cells with the nucleoside analog or the mitochondrial inhibitor as single agents or in combination (Fig. 5). We found that melanoma cells lacking NRAS or BRAF mutations (WT/WT) were resistant to 6-thio-dG and Gamitrinib as single agents or in combination, as these compounds did not induce cell death (Fig. 5a). As we have previously shown, BRAF-mutant melanoma cells were highly sensitive to Gamitrinib [39]; whereas Gamitrinib alone led to 75–80% cell death, the combination with 6-thio-dG did not further enhanced death of BRAF-mutant melanoma cells (Fig. 5b). As the aforementioned data indicated that Gamitrinib selectively potentiates the efficacy of 6-thio-dG in NRAS-mutant melanoma cells, we wondered whether this combination could trigger cell death in other RAS-mutant tumor cells. To answer this question, we tested the effects of Gamitrinib and 6-thio-dG in KRAS-mutant lung and colon cancer cell lines as well as NRAS-mutant glioblastoma cells. Interestingly, 6-thio-dG and Gamitrinib triggered significant death of NRAS-mutant SKNAS glioblastoma cells, as single agents or in combination (Fig. 5c). In contrast, 6-thio-dG and Gamitrinib did not induce substantial death of KRAS-mutant HCT116 and A549 cells (Fig. 5d, e). These data suggest that Gamitrinib potentiates the efficacy of 6-thio-dG selectively in NRAS-mutant tumor cells.

**Fig. 6:**
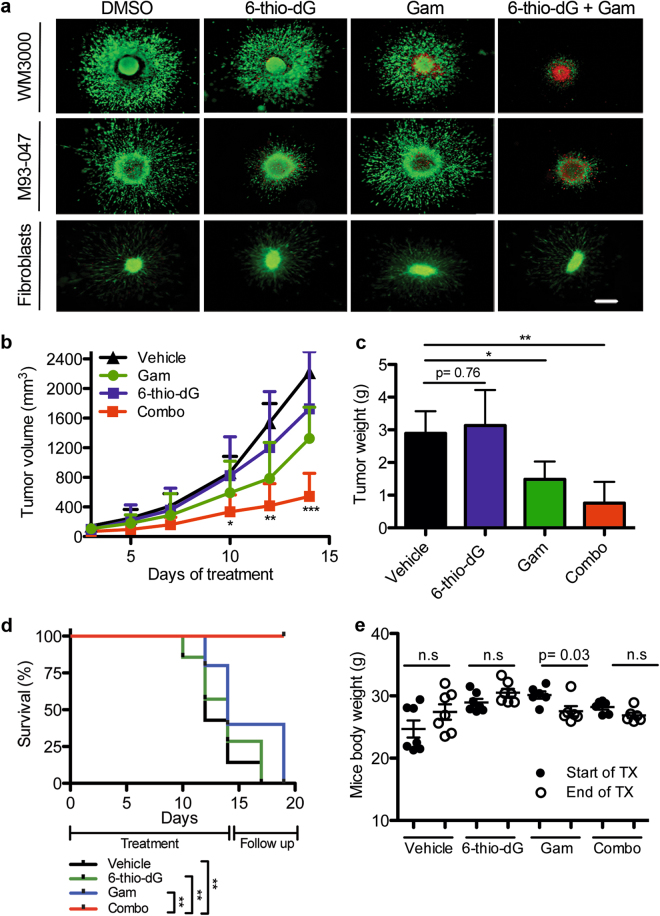
Combining 6-thio-dG and Gamitrinib inhibits the growth of 3D melanoma spheroids and NRAS-mutant xenograft tumors. **a** Collagen-embedded melanoma spheroids were treated with DMSO, 6-thio-dG (5 µM), or Gam (5 µM) as in Fig. 4. On day 7, spheroids were stained with Calcein AM (live cells; green) and EtBr (dead cells; red), and imaged using an inverted microscope (×4; scale bar = 250 µm). Representative merged images from three independent experiments are shown. **b**–**e** NRAS-mutant M93-047 tumor-bearing mice were treated with vehicle control, 6-thio-dG (2.5 mg/kg; ip qd), Gam (12.5 mg/kg; ip qod), or the combination of the two drugs for the indicated days (*n* = 7 mice/group). **b** Average tumor volume over time ± SEM is represented. **c** Average tumor weight ± SEM after 14 days of treatment. *p-*values were calculated using unpaired Student’s *t*-test. *p < 0.05, **p < 0.01, ***p < 0.005, when comparing vehicle control vs. combination treatment groups. **d** Treatment was discontinued after 14 days, and mice were followed until tumor volume reached a preset volume (1500 mm^3^). Kaplan–Meier survival curves of mice treated for 14 days. *p*-values were calculated by Mantel–Cox log-rank test; *p < 0.05, **p < 0.01, ***p < 0.005. **e** Animal weight (grams) before treatment (start of TX) and at the end of the study (end of TX) was assessed and recorded for every mouse enrolled in the study. Mean and SEM are depicted (*n* = 7 mice per treatment group). Statistical significance was assessed by unpaired Student’s *t*-test

To further evaluate the efficacy of 6-thio-dG in combination with Gamitrinib, we treated NRAS-mutant melanoma cells grown as 3D-collagen-embedded spheroids, which more closely mimic the in vivo behavior of melanoma. Pre-treatment of NRAS-mutant melanoma spheroids with 6-thio-dG markedly potentiated the effect of Gamitrinib, and induced apoptosis of 3D melanoma spheroids (Fig. 6a). Of note, although treatment with another mitochondrial inhibitor phenformin resulted in similar cooperativity with 6-thio-dG (Supplementary Figure 10a), treatment with ganetespib, an inhibitor of cytosolic HSP90, did not substantially enhance the efficacy of 6-thio-dG (Supplementary Figure 10b). These data support the premise that drug-induced mitochondrial impairment potentiates the effects of 6-thio-dG.

**Fig. 7:**
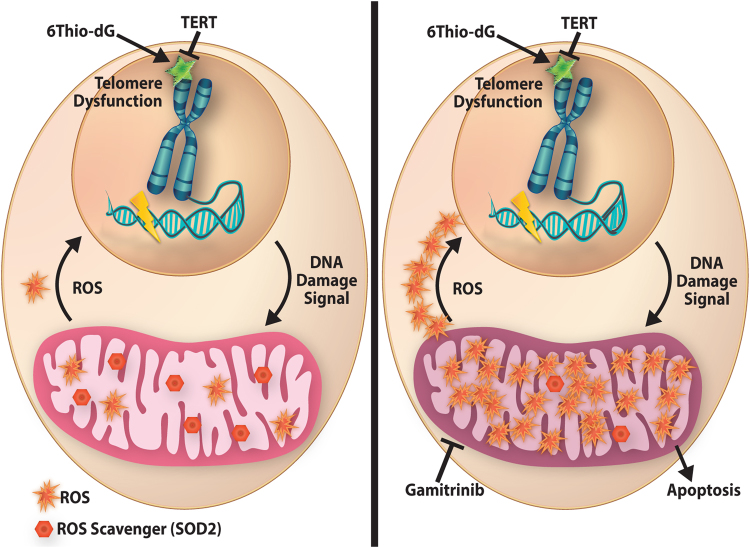
TERT offsets oxidative stress in NRAS-mutant melanoma. TERT is required for telomere integrity. Genetic silencing of TERT or pharmacological induction of telomere uncapping due to incorporation of the nucleoside analog 6-thio-dG triggers rapid telomere-induced foci (TIF) coupled to global DNA damage and increased production of ROS. Enhanced ROS levels prompts the activation of a ROS-scavenging adaptive response, mediated mainly by SOD2, which re-establishes steady levels of ROS and offsets oxidative stress. Treatment of NRAS-mutant melanoma cells with Gamitrinib, a mitochondrial disrupting agent, attenuates SOD2 levels impairing its ability to effectively restore redox balance, resulting in toxic levels of ROS and tumor cell death

Furthermore, mice treated with the combination of 6-thio-dG plus Gamitrinib exhibited significantly smaller tumors, compared to mice treated with either single agent (Fig. 6b, c). The combination of 6-thio-dG plus Gamitrinib substantially prolonged the survival of tumor-bearing mice (Fig. 6d), even after treatment withdrawal. Of note, this combination was well tolerated in vivo and did not significantly affect animal weight (Fig. 6e). Together, our results indicate that therapeutic approaches exploiting melanoma cell addiction to TERT, and combating the ability of NRAS-mutant melanoma to offset oxidative stress, would render cancer cells vulnerable to drug-induced cell death (Fig. 7).

## Discussion

The discovery of TERT promoter mutations in most melanomas has provided compelling evidence that this gene has a critical role in this disease, and, therefore, constitutes a promising therapeutic target. Here we used NRAS-mutant melanoma as a treatment refractory model to investigate the therapeutic value of exploiting melanoma’s addiction to TERT, and activation of adaptive mechanisms limiting the effects of TERT-based approaches. We found that telomere-induced dysfunction is associated with oxidative damage followed by relatively rapid melanoma cell death in vitro and decreased tumor growth in vivo. However, tumor cells counteract these effects by activating an antioxidant program aimed at restoring redox balance. We show that impairing mitochondrial function and blunting the ROS-scavenging machinery renders NRAS-mutant tumor cells susceptible to excessive ROS levels, leading to tumor cell death.

Despite the critical role of telomerase in cancer, developing effective anti-telomerase therapies has been challenging [14, 40]. Only one compound, imetelstat (GRN163L), an oligonucleotide that binds to the RNA subunit of telomerase TERC, has shown efficacy in myeloproliferative disorders, but has displayed limited activity in solid tumors [14, 41, 42]. Preclinical approaches to inhibit telomerase have been evaluated in melanoma; nevertheless, telomerase inhibitors have shown modest activity as monotherapy and they have not yet been successfully translated to the clinical setting [18, 43, 44]. Interestingly, inactivation of p21 combined with treatment with imetelstat and CP-31398 (to restore p53 activity), repressed melanoma growth [45], suggesting that telomerase-based combination approaches might lead to enhanced anti-tumor effects.

The lack of success developing effective anti-telomerase approaches and the reason why solid tumors respond poorly to telomerase monotherapy remains elusive. This could be in part related to early efforts focusing mainly on targeting the reverse transcriptase activity of telomerase and being heavily reliant on telomere shortening. A shortcoming of this approach is the relatively long lag period needed for efficacy, which could trigger activation of adaptive mechanisms and drug resistance. In our studies, we found that either TERT silencing or treatment with 6-thio-dG led to telomere dysfunction and relatively rapid cell death. Even though we did not detect significant changes in average telomere length within the time frame that cells underwent apoptosis, it is possible that critically short telomeres, commonly present in melanoma cells, become uncapped and dysfunctional, thereby also contributing to the cell death. Of note, it has been reported that melanoma cells harbor a subset of critically short telomeres and that the upregulation of telomerase activity associated with TERT promoter mutations does not preclude telomere attrition [16, 46, 47]. In fact, Chiba and colleagues recently demonstrated that reactivation of telomerase via TERT promoter mutations allows for melanomagenesis first by protecting the shortest telomeres rather than by elongating the telomeres, and subsequently by sustaining tumor cell proliferation. These studies and our results suggest that anti-melanoma strategies dependent solely on telomere shortening may not have a significant impact on tumor maintenance [48]. In contrast, induction of telomere uncapping and dysfunction appears to have a more rapid effect triggering apoptosis in a telomere length-independent manner [36, 49]. In support of this premise, Blasco and colleagues [49] recently showed that induction of acute telomere uncapping by depletion of the shelterin protein, TRF1, can restrain tumor growth independently of telomere length in a p53-null KRas (G12V)-induced lung carcinoma mouse model. Likewise, it has been shown that ectopic expression of telomerase can protect cells against double-strand DNA damage in a telomere length-independent manner [50]. We found that non-toxic doses of the telomere uncapping agent, 6-thio-dG, as a single agent slowed, but did not completely abrogated tumor growth in vivo, raising the possibility that induction of telomere dysfunction could prompt the activation of adaptive or compensatory survival mechanisms mitigating the effects of the drug.

Although resistance to TERT-based approaches has been attributed primarily to the engagement of alternative lengthening of telomeres (ALT) [51, 52], little is known about adaptive survival mechanisms triggered by TERT inhibition or telomere dysfunction. We found that although telomere dysfunction is associated with oxidative damage, melanoma cells rapidly activate an antioxidant response coupled to increased expression of PGC-1α and ROS-scavenging enzymes, primarily Mn-superoxide dismutase SOD2 with no evidence of ALT activation. These results led us to postulate that a rational strategy could involve combining 6-thio-dG with agents that further induce oxidative stress and disable the antioxidant machinery of tumor cells. We selected Gamitrinib, a mitochondriotoxic small molecule, that selectively blocks mitochondrial HSP90 and exhibits broad anti-cancer activity including efficacy in BRAF-mutant melanoma, but limited activity in NRAS-mutant cells as a single agent [39]. Notably, melanoma drug resistance to BRAF inhibitors in BRAF-mutant melanoma can be mediated by mitochondrial adaptive responses [53–55]. Likewise, Hu et al. [51] demonstrated in an ATM-deficient lymphoma mouse model that genetic ablation of telomerase caused cell death, but also induced ALT and PGC-1β. They further showed that genetic depletion of PGC-1β impaired mitochondria function, enhancing anti-telomerase therapy. Altogether, these studies suggest that mitochondrial function might have a major role modulating multi-drug response and cancer cell viability.

The exact mechanism triggering the mitochondrial detoxifying response prompted by TERT depletion or treatment with 6-thio-dG needs to be further investigated. Several lines of evidence suggest that TERT has other roles independent of its canonical telomere-lengthening function [20]. For example, TERT has been shown to traffic to mitochondria [56, 57]; however, the relevance of mitochondrial TERT is not fully understood. Previous reports suggest that ROS can modulate TERT intracellular localization and that TERT can bind to mitochondrial DNA, promoting resistance to oxidative stress and increasing cell survival [58]. Consistent with our studies, TERT overexpression attenuates ROS basal levels and diminishes stressed-induced ROS generation [59]. We found that both TERT depletion or treatment with 6-thio-dG upregulated ROS, and that TERT can potentiate the antioxidant capacity of melanoma cells in a RT and telomere length-independent manner, enabling melanoma cells to survive under conditions of excessive oxidative stress. As ROS signaling can induce the expression of FOXO transcription factors, FOXO along with the transcriptional co-activator PGC-1α could enhance the expression of detoxifying enzymes genes such as SOD2 in NRAS-mutant melanoma cells [60, 61].

Our studies support the notion that NRAS-mutant melanoma is a prime candidate for TERT-based therapeutic approaches. Our data suggest that in addition to its cannonical role, TERT may also possess a telomere length-independent role promoting melanoma survival, as catalytically impaired and telomere elongation-deficient TERT can protect cells from loss of oncogenic NRAS. These results could have therapeutic implications, as they raise the possibility that approaches solely targeting the reverse transcriptase activity of TERT could have limited efficacy impairing tumor growth and maintenance.

Altogether, our studies stress the need to develop drug combinations co-targeting not only telomerase’s catalytic activity but also other telomere length-independent functions and adaptive-resistant mechanisms. Our data provide proof-of-principle for this strategy and for developing similar combinations to increase anti-tumor responses. Finally, it would be important in future studies to further determine whether this approach could also be applied to other NRAS-driven tumors.

## Material and methods

### Cell culture, viability, and cell death assays

All cells were cultured in RPMI-1640 medium (Corning Cellgro, Manassas, VA) supplemented with 5% fetal bovine serum (FBS) and grown at 37 °C in 5% CO_2_. Human fibroblasts (FF2511) were isolated from foreskin samples and grown in RPMI-1640 supplemented with 10% FBS.

Cells were seeded in 96-well plates and treated with drugs. 6-thio-dG was purchased from R.I. Chemical Inc (Orange, CA). The complete chemical synthesis, HPLC profile, and mass spectrometry of mitochondrial-targeted small molecule HSP90 antagonist, Gamitrinib (GA mitochondrial matrix inhibitors) has been reported [33]. The Gamitrinib variant containing triphenylphosphonium as a mitochondrial-targeting moiety was used in this study. Cell viability was assessed following 6 h incubation with 500 µM Alamar Blue (ThermoFisher Scientific, Waltham, MA) using an EnVision Xcite Multilabel plate reader (Perkin Elmer, Waltham, MA). Cell death was determined by flow cytometry using PSVue-643 (p-1006; Molecular Targeting Technologies, West Chester PA) or Annexin V (640919; Biolegend, San Diego, CA) and Propidium Iodide (Sigma-Aldrich, St. Louis, Mo) staining. Samples were analyzed using a BD LSRII flow cytometer (BD Biosciences, San Jose, CA) and analyzed using FlowJo Software v10.0.7 (FlowJo, LLC, Ashland, OR, USA). Analysis of samples by flow cytometry was performed blindly.

### TERT constructs, small hairpin RNA, and lentivirus infection

Lentiviral NRAS shRNA in pLKO1 backbone and TERT shRNA in pGIPZ backbone were obtained from ThermoFisher Scientific. TERT constructs (Wild-type, FVYL1016, or FVYL1028) in a pBlast lentiviral vector have been previously described [35]. Lentiviruses were produced by transfection of 293T cells with packaging plasmids (pPAX2 and pMD2.G) along with 4 µg lentiviral shRNA vector using Lipofectamine 2000 reagent (Invitrogen, Waltham, MA) following the manufacturer’s instructions. Melanoma cells were transduced with virus in the presence of 8 µg/ml polybrene (Sigma-Aldrich) for 18 h. Transduced cell populations were selected with appropriate antibiotics. shRNA knockdown efficiency was determined by western blot analysis and/or qRT-PCR.

### PCR array

Human cellular senescence RT2 Profiler PCR array (Qiagen, Valencia, CA) was used following manufacturer’s specifications. Data was analyzed with the SABiosciences PCR Array Data Analysis Template Excel.

### Immunobloting

For western blot analysis, total cell lysates were prepared as previously described [62]. Nitrocellulose membranes were incubated overnight with primary antibodies at 4 °C, followed by 1 h incubation with Alexa Fluor-labeled secondary antibodies (IRDye 680LT goat-anti mouse or IRDye 800CW goat anti-rabbit antibodies (LI-COR Biosciences)) at room temperature. Fluorescent images were acquired and quantified by LI-COR Odyssey Imaging System. Antibodies used are detailed in Supplementary materials and methods section.

### Telomerase activity assay

Telomerase activity was measured by TRAP assay as previously described [31]. HCT116 cells and lysis buffer were used as positive and negative controls, respectively. Telomerase extension products were amplified by PCR and run on 10% non-denaturing acrylamide gel. Typhoon Phosphoimager scanner (Molecular Dynamics, GE Healthcare, Little Chalfont, UK) was used for visualization of gel products.

### Telomere length assay

Genomic DNA was prepared using Wizard genomic DNA purification kit (Promega) following manufacturer’s instruction. For telomere length and Southern blot analysis, genomic DNA (~5 μg) was digested with AluI + MboI restriction endonucleases, fractionated in a 0.7% agarose gel, denatured, and transferred onto a GeneScreen Plus hybridization membrane (PerkinElmer). The membrane was cross-linked, hybridized overnight at 42 °C with 5′-end-labeled 32P-(TTAGGG)4 probe in Church buffer (0.5 N Na_2_HPO_4_ pH 7.2, 7% SDS, 1% BSA, 1 mM EDTA), and washed twice for 5 min each with 0.2 N wash buffer (0.2 N Na_2_HPO_4_ pH 7.2, 1 mM EDTA, and 2% SDS) at room temperature and once for 10 min with 0.1 N wash buffer at 42 °C. The images were analyzed with Phosphorimager, visualized by Typhoon 9410 Imager (GE Healthcare), and processed with ImageQuant 5.2 software (Molecular Dynamics).

### DNA damage and telomere dysfunction assay (TIF)

For assessment of global DNA damage cells were fixed and permeabilized with BD CytoFix/Perm reagent following the manufacturer’s instructions and incubated with γH2AX antibody (Cell Signaling Technology, Danvers, MA) for 1 h at room temperature followed by 1 h incubation with Alexa-647 anti-Rabbit secondary antibody (A21244; Life Technologies, Carlsbad, CA). Mean fluorescence staining was quantified by flow cytometry. For NAC treatment, cells were treated with 1 mM *N*-acetyl-*N*-cysteine (Sigma-Aldrich) in PBS for the duration of the experiment. NAC was replaced every 48 h up to day seven.

### Measurement of reactive oxygen species

General or mitochondrial specific ROS were measured by flow cytometry H2DCFDA or MitoSoxRed (Invitrogen, Waltham, MA) following the manufacturer’s specifications.

### 3D tumor spheroid models

Cells 5 × 10^3^ were seeded in 96-well plates coated with 1% agar in PBS and allowed to grow for 72 h. Spheroids were embedded into rat collagen type I (Corning, Bedford, MA) mixture as previously described [62] and treated with drugs for 7 days. Spheroids were stained with Live/Dead cell assay (Invitrogen, Waltham, MA) and imaged using a Nikon Inverted TE2000 microscope (Melville, NY, USA). Images were processed and merged using Image Pro software (Media Cybernetics, Rockville, MD, USA).

### Animal studies

Female and male (5–6 weeks old) NOD/LtSscidIL2Rγ-null mice (NSG) mice were injected subcutaneously with 1 × 10^6^ NRAS^mut^ melanoma cells (WM3000 or M93-047) in a suspension of matrigel (BD Matrigel Basement Membrane Matrix, Growth Factor Reduced)/RPMI media at a ratio of 1:1. Tumor growth was measured twice weekly with digital calipers. Once tumors reached an average volume of 50–100 mm^3^, mice were randomized into different treatment groups. Randomization was performed for all in vivo studies using Random.org following atmospheric noise algorithm. For single drug studies, 2.5 mg/kg of 6-thio-dG was administered (i.p, q.d.). For combination studies, same dose of 6-thio-dG was administered alone for the first 7 days (q.d.). Mice were then treated with 12.5 mg/K of Gamitrinib (i.p, q.d.) and 6-thio-dG (ip, q.o.d.) up to 20 days. Tumor volume over time were used to model tumor growth rate in each treatment group. No blinding was done for these studies.

To have at least 80% power with a two-sided type I error rate of 5%, 7 mice per group were used for in vivo drug combination studies. The only criteria for exclusion was health and well-being of the animals. No animals were excluded from the analysis.

Tumor growth rates were compared between treatment groups using a linear mixed-effect model or mixed-effect spline model with the random effect at individual animal level. A *p*-value < 0.05 was considered statistically significant. For survival studies, treatment was discontinued after 20 days, and animals were followed-up for 10 additional days. For survival analysis, tumor volume endpoint was preset at 1500 mm^3^ and data represented as Kaplan–Meier curves.

All animal studies were approved by the Wistar Institute IACUC. All animal studies were conducted in accordance with NIH animal care and use guidelines, and mice were maintained according to the guidelines of the IACUC of The Wistar Institute.

### Statistical analysis

All experiments were performed at least three independent times unless otherwise indicated; sample size (*N*) is indicated in the figure legends. Data are expressed as average ± SEM unless otherwise indicated. For in vitro experiments with *n* = 3 per group, we target 80% power to test a large effect size of 3.1 at a two-sided type I error rate of 5%. Results normally distributed with equal variance between groups were analyzed by unpaired two-tailed Student’s *t*-test. If variance was not similar between groups, Student’s *t*-test with unequal variances was applied. Asterisks denote *p*-value significance: *p < 0.05; **p < 0.01; ***p < 0.005. Sample sizes, statistical tests, and *p-*values are indicated in the figure legends. All statistical analyses were calculated using Stata 14, GraphPad Prism5 software, or Microsoft Excel. For microscope images (spheroids, IF) and immunoblots representative images of three independent experiments are shown.

## Electronic supplementary material

Supplementary Figures 1-10

Supplemental material
