# Supplementary material for: Exploiting TERT dependency as a therapeutic strategy for NRAS-mutant melanoma
Source: Oncogene. 2018 Apr 26;37(30):4058–72. doi: 10.1038/s41388-018-0247-7 (PMC6062502; doi:10.1038/s41388-018-0247-7)
Supplement: Supplementary file 1 — Supplementary Figures 1-10 [file 41388_2018_247_MOESM1_ESM.pdf]

SUPPLEMENTARY FIGURES

EXPLOITING TERT DEPENDENCY AS A THERAPEUTIC STRATEGY FOR NRAS MUTANT MELANOMA

Patricia Reyes-Uribe [1,2], Maria Paz Adrianzen-Ruesta [1,2], Zhong Deng [1,3], Ileabett Echevarria-Vargas [1,2], Ilgen Mender[5], Steven Saheb [1,2], Qin Liu [1,2], Dario C. Altieri [1,4], Maureen E. Murphy[1,2], Jerry W. Shay[5], Paul M. Lieberman[1,3], and Jessie Villanueva[1,2]*.

The Wistar Institute[1], Molecular & Cellular Oncogenesis[2], Gene Expression & Regulation[3], and Immunology, Microenvironment and Metastasis[4] Programs, Philadelphia, PA 19104. University of Texas Southwestern Medical Center, Department of Cell Biology, Dallas, TX 75930-9039[5]

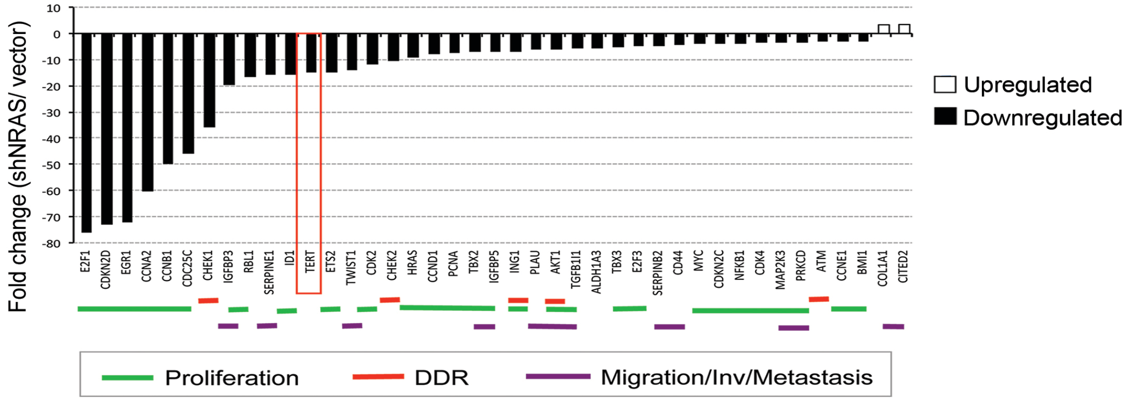

Supplementary Figure 1. NRAS silencing is coupled to downregulation of TERT.

NRAS-mutant WM3000 cells transduced with lentiviruses encoding NRAS shRNA were analyzed using the human cellular senescence PCR array profiler. mRNA levels of the indicated genes were assessed by qRT-PCR following NRAS knockdown.

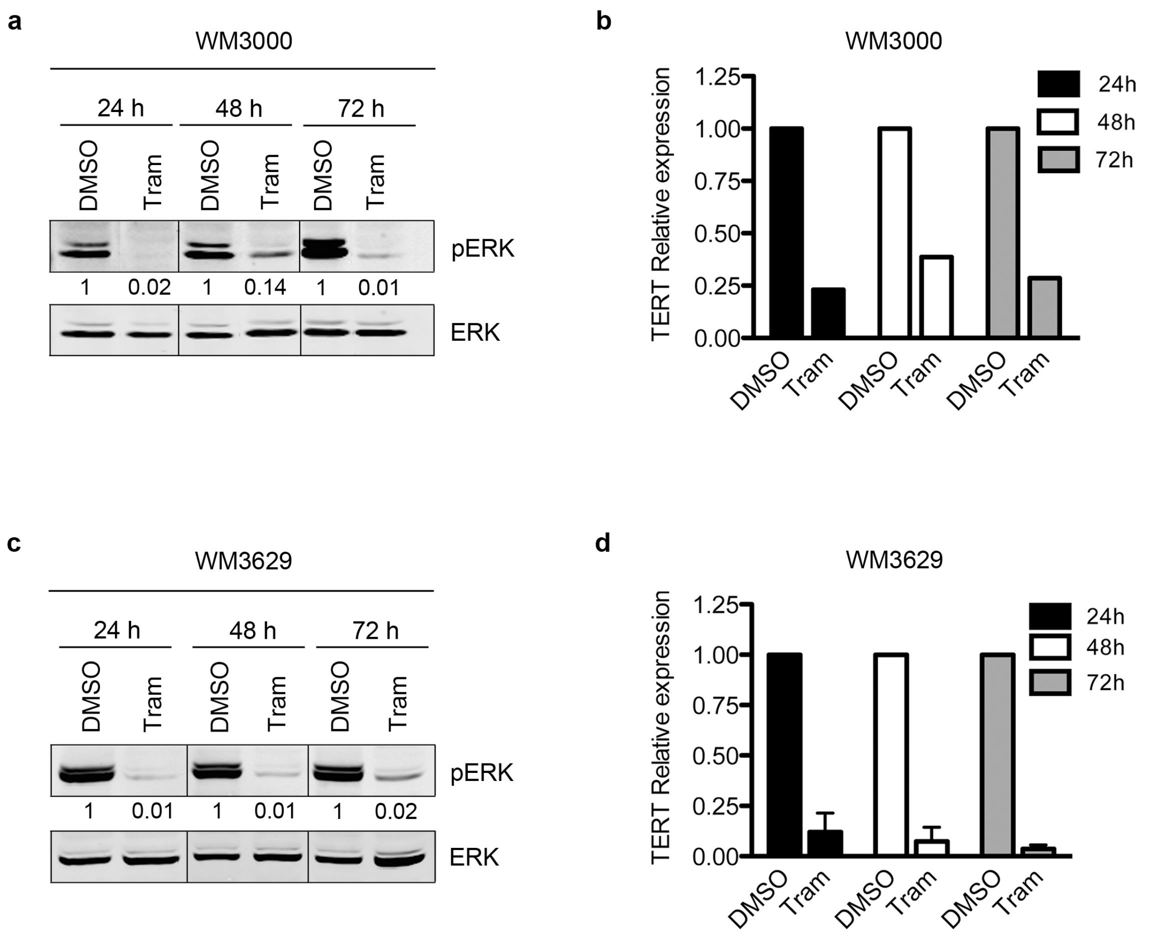

Supplementary Figure 2. MEK inhibition downregulates TERT levels.

NRAS mutant melanoma cells WM3000 (a, b) or WM3629 (c, d) were treated with the MEK inhibitor trametinib (100 nM) for 24, 48, 72h. Inhibition of phospho-ERK was assessed by immunoblotting. Dotted lines indicated where membranes were cut to remove non-relevant lanes (a, c). Relative expression of TERT mRNA levels was determined by qRT-PCR (b, d). Data represent average of triplicates +/- SD.

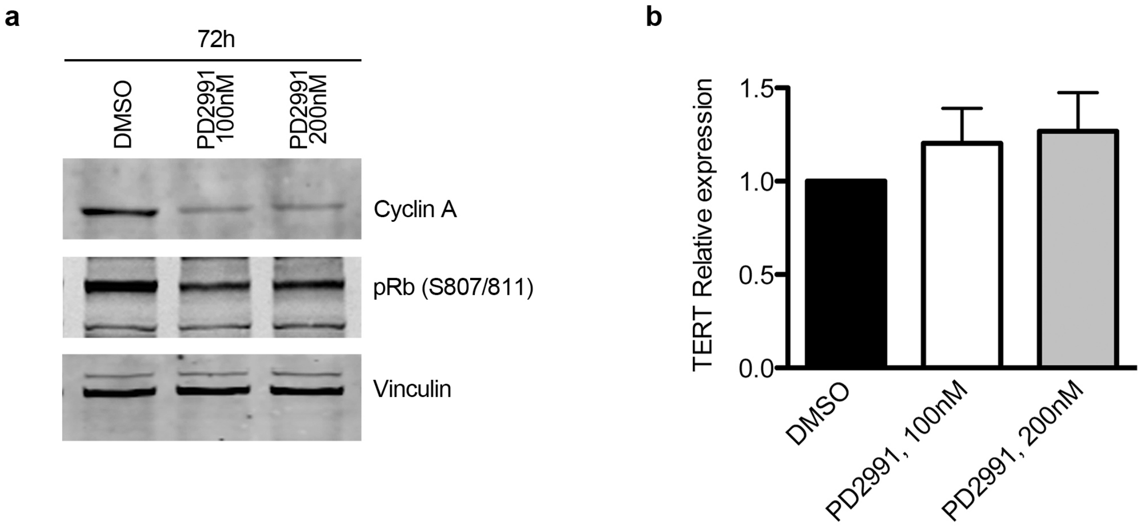

Supplementary Figure 3. Inhibition of cdk4/6 transiently attenuates TERT expression.

NRAS mutant melanoma cells WM3000 were treated with the cdk4/6 inhibitor palbociclib at the indicated doses for 72h. (a) Downregulation of phospho-Rb and cyclin A was evaluated by immunoblotting as a surrogate of cell cycle arrest. (b) Relative expression of TERT mRNA levels was determined by qRT-PCR. Data represent average of triplicate samples.

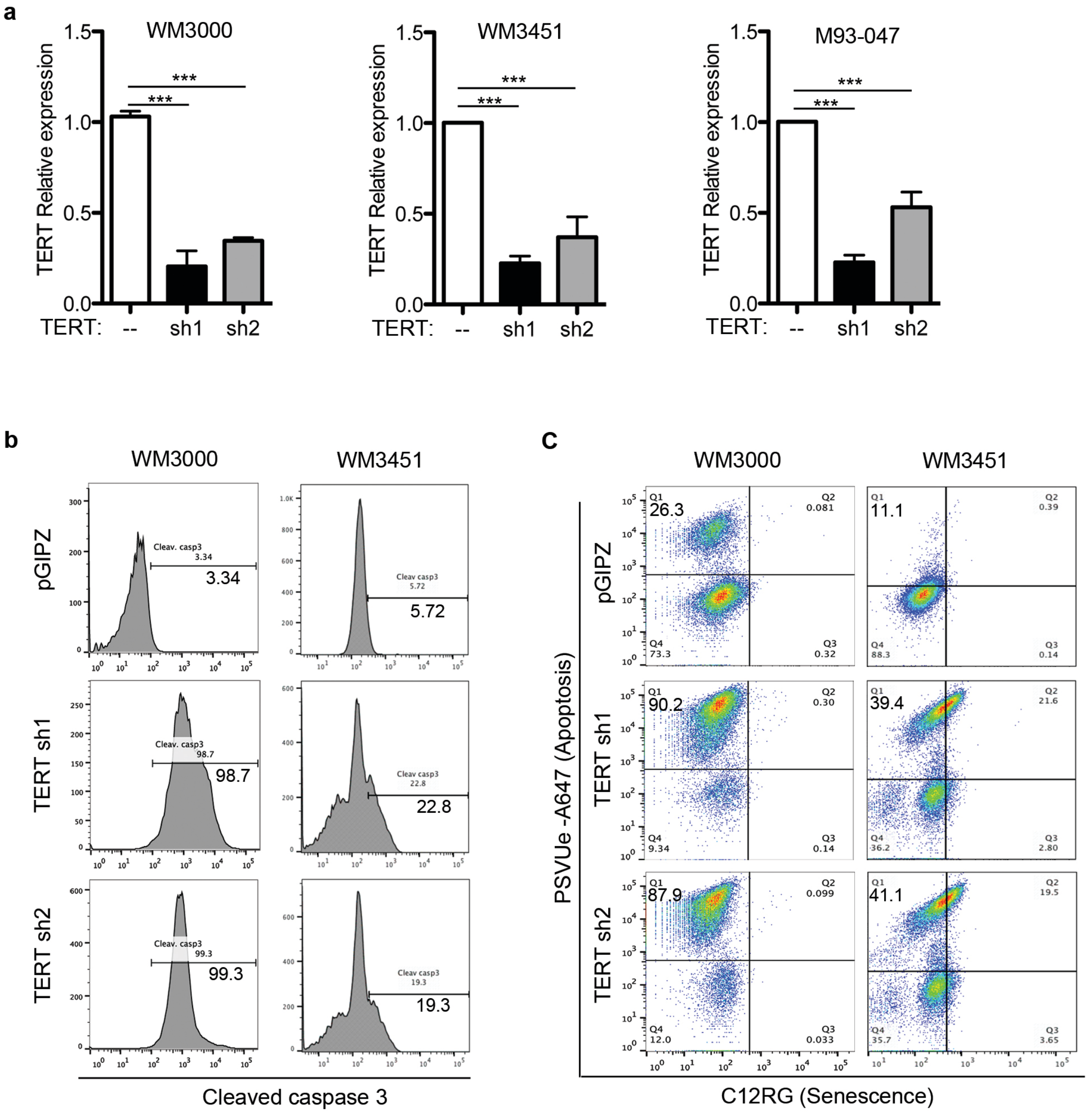

Supplementary Figure 4. TERT depletion triggers apoptosis.

(a-c) NRAS^{mut} melanoma cell lines were transduced with lentiviruses encoding TERT shRNA using two different hairpins (sh1, sh2) or non-targeting empty vector pGIPZ and analyzed 11 days post infection (dpi). Data shown represent average of three independent experiments +/- SEM. TERT mRNA levels were assessed by qRT-PCR (a). Apoptosis was determined using cleaved caspase 3 by FACS (b). Apoptosis was also assessed by using the Annexin V analog

PSVue and senescence was assessed using the ImaGene Red β-galactosidase substrate C12RG by FACS (c).

a

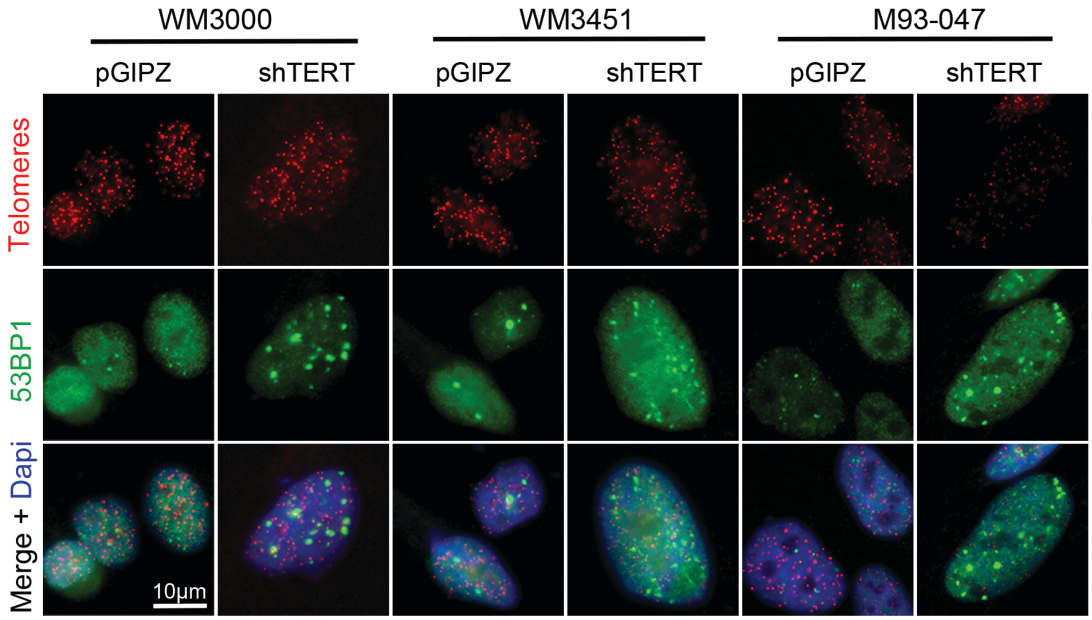

c

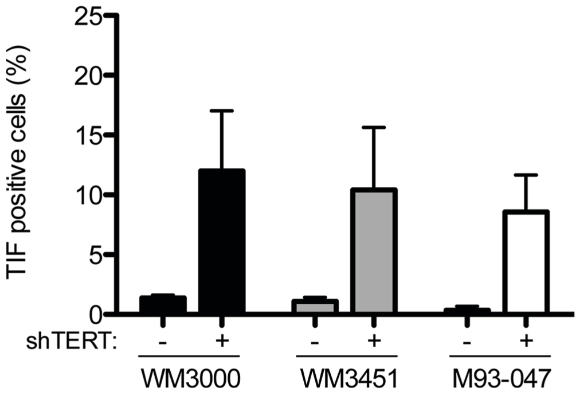

Supplementary Figure 5. TERT silencing triggers rapid telomere dysfunction-induced foci.

(a) Telomere induced foci (TIF) was determined by indirect immunofluorescence combined with fluorescence in situ hybridization (FISH) in NRAS^{mut} cells transduced with TERT shRNA 7dpi. Cells were considered TIF positive when four or more co-localizing foci of telomeres (red) and 53BP1 (green) were found.

(b) Quantification of TIF positive cells from two independent experiments. Data are average of two independent experiments +/- SEM.

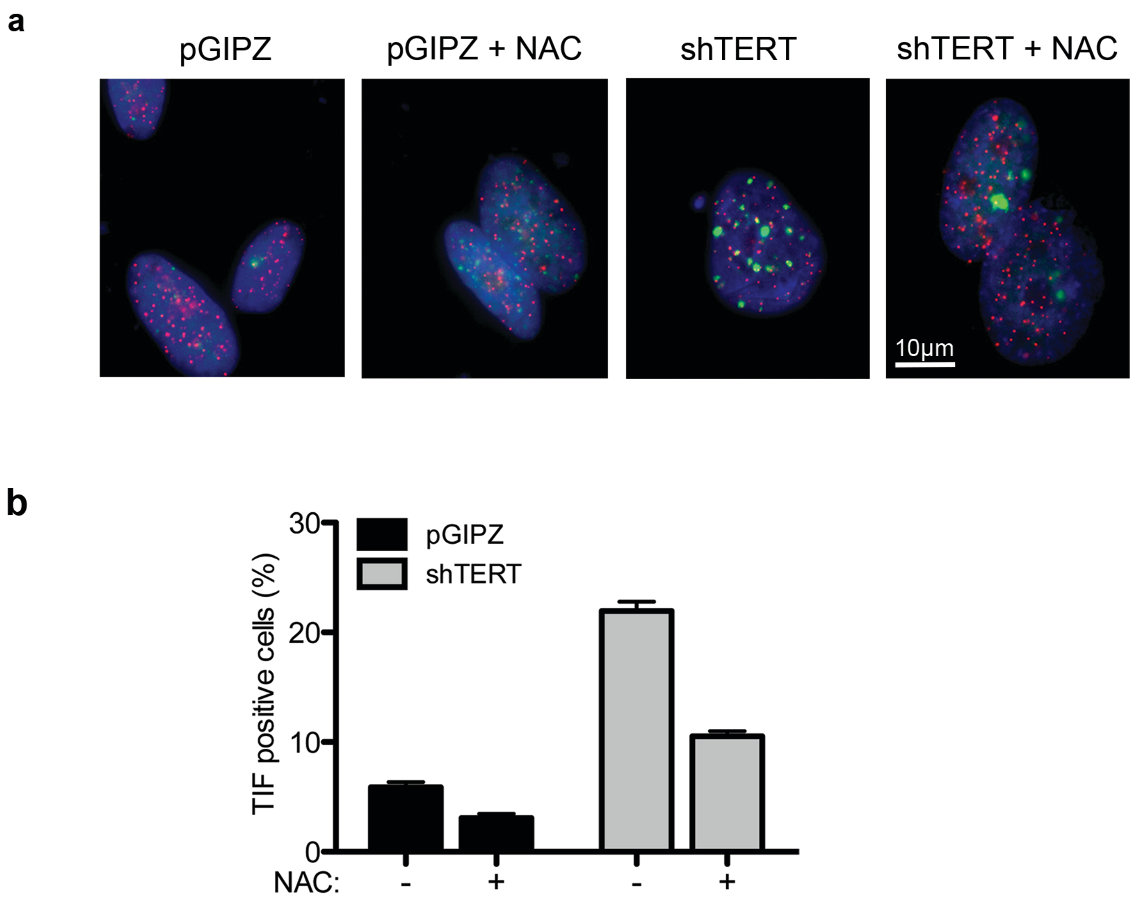

Supplementary Figure 6. Treatment with the antioxidant N-acetyl-L-cysteine (NAC) attenuates TIFs.

NRAS mutant melanoma cells WM3000 were transduced with TERT shRNA and treated with NAC (mM) for 7 days. (a) TIFs were assessed by indirect immunofluorescence combined with fluorescence in situ hybridization (FISH). Representative images are shown. (scale bar=10 μm). (b) Quantification of TIF positive cells. Cells were scored as TIF positive when three or more γ-H2AX foci (green) were co-localizing with telomere foci (red). Data represent average of thirteen fields imaged +/-SD.

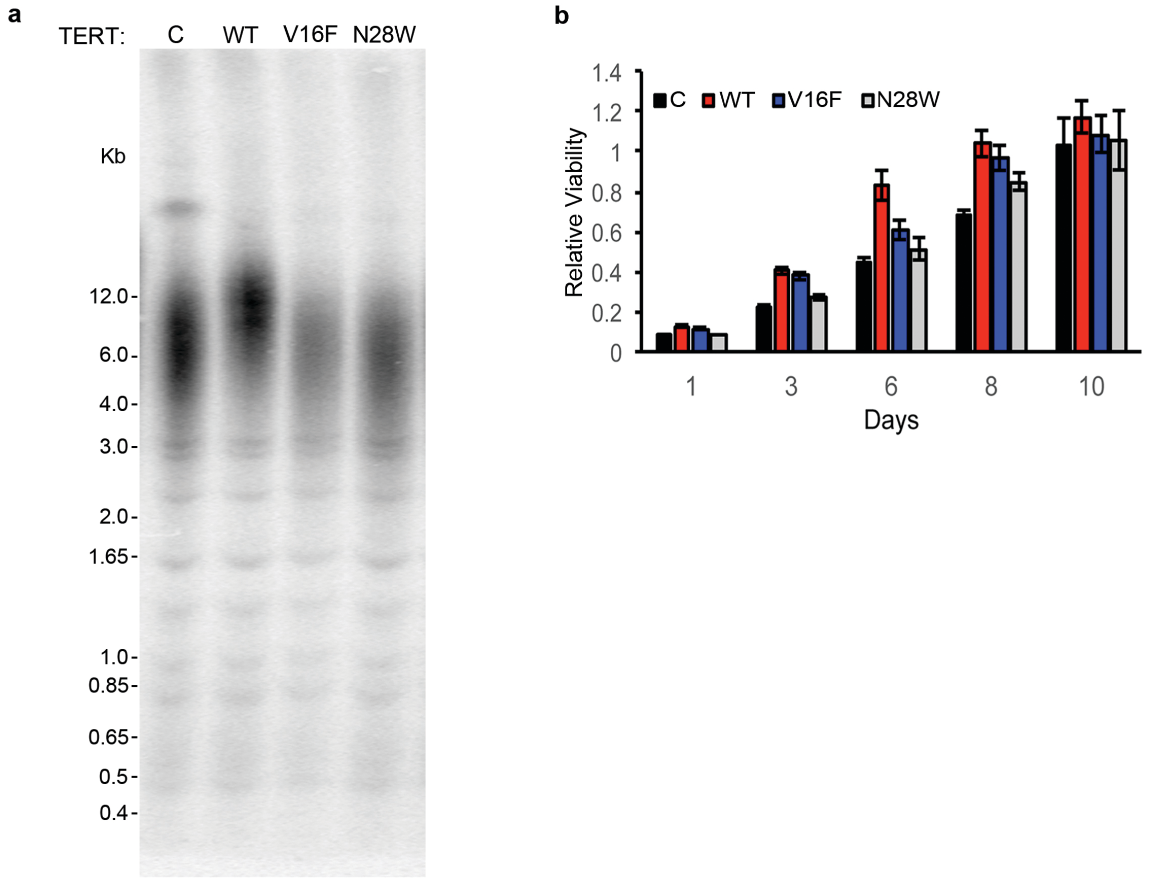

Supplementary Figure 7. Catalytically impaired TERT mutants lead to telomere attrition in NRAS mutant melanoma cells.

(a) Telomere length was assessed by Southern blotting of terminal restriction fragments (TRF) in WM3000 cells transduced with vector control, wild type (WT), or catalytic-impaired hTERT mutant constructs VYLF1016 (V16F) or VYFL1028 (N28W). (b) Relative cell viability was determined by MTT assays at days 1, 3, 6, 8 and 10 post-transduction. Cell viability was calculated relative to vector control-transduced cells.

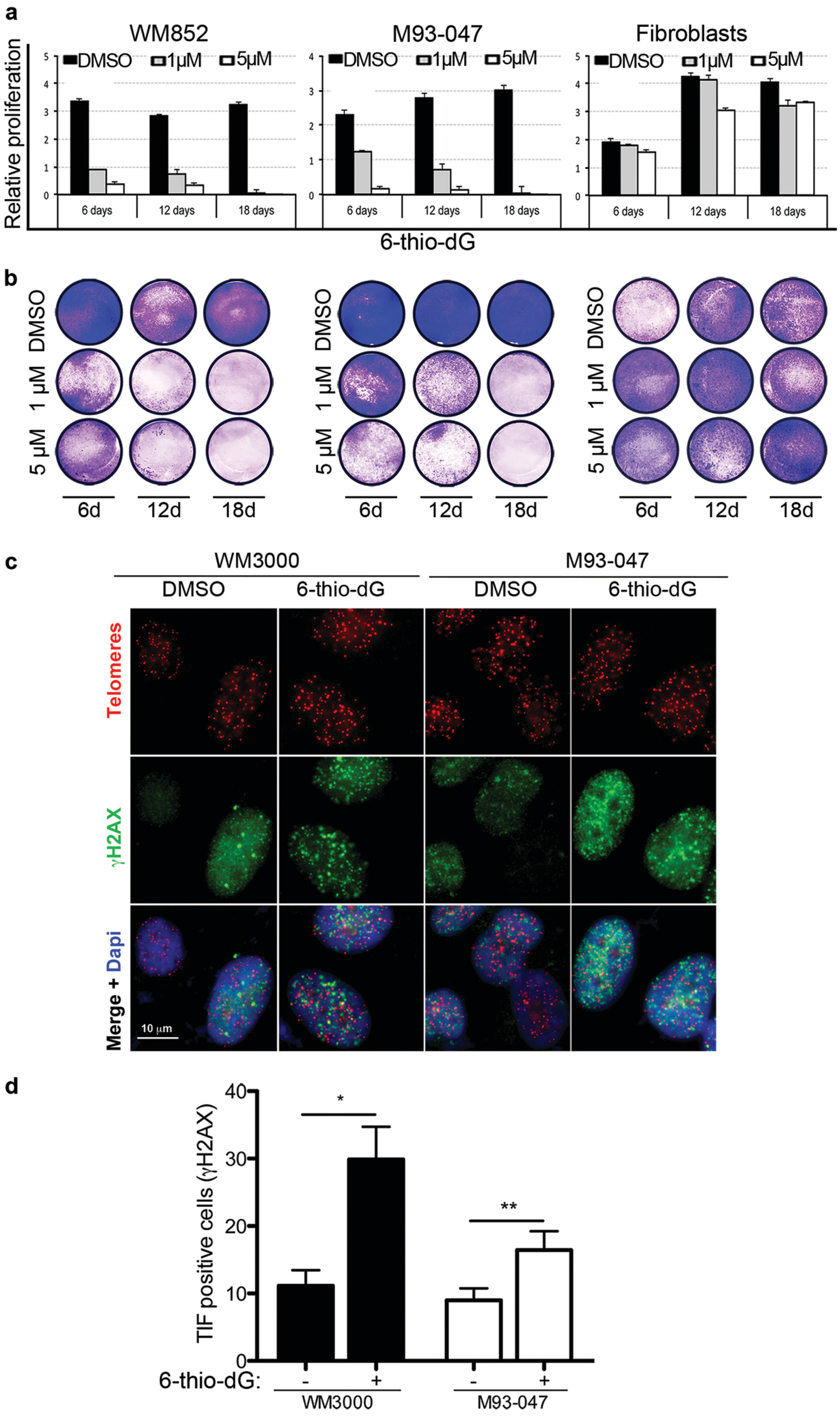

a
WM852
M93-047
Fibroblasts
DMSO 1μM 5μM
Relative proliferation
6 days
12 days
18 days
6-thio-dG
b
DMSO
1 μM
5 μM
6d
12d
18d
c
WM3000
M93-047
DMSO
6-thio-dG
Telomeres
γH2AX
Merge + Dapi
10 μm
d
TIF positive cells (γH2AX)
6-thio-dG:
WM3000
M93-047

Supplementary Figure 8. NRAS mutant melanoma cells are sensitive to the telomere uncapping agent 6-thio-dG.

$NRAS^{mut}$ melanoma cells were treated with 6-thio-dG for 6, 12 or 18 days. (a) Relative cell number was assessed by Crystal Violet assay and calculated relative to DMSO-treated cells. Data represent average of three independent experiments +/- SD. (b) Representative pictures of cells treated with 6-thio-dG for the indicated days and stained with Crystal Violet. (c) $NRAS^{mut}$ cells were treated with 6-thio-dG for 7 days and telomere induced foci (TIF) was determined by indirect immunofluorescence combined with fluorescence in situ hybridization (FISH). Cells were considered TIF positive when four or more co-localizing foci of telomeres and γ-H2AX were found. (d) Quantification of TIF assay. Cells were scored as TIF positive when four or more γ-H2AX foci (green) were co-localizing with telomere foci (red). Data represent average of two independent experiments +/- SEM. P values were calculated by unpaired Student's t-test.

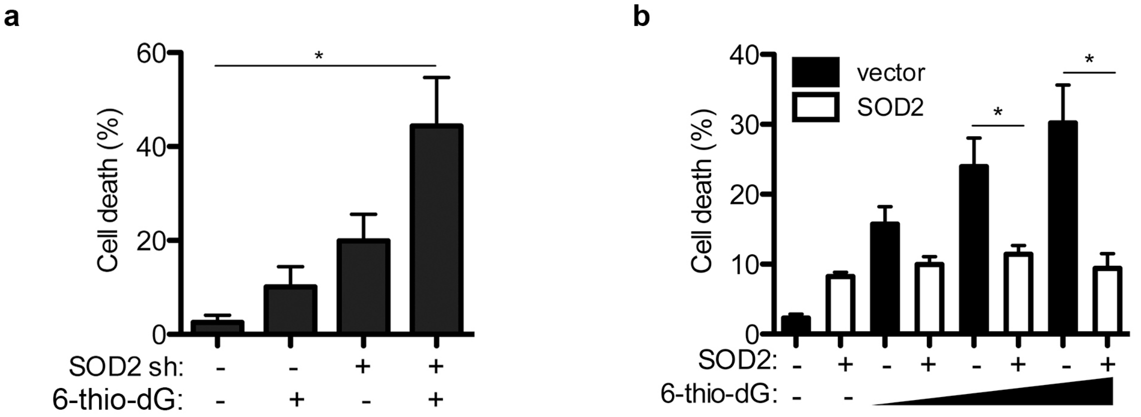

Supplementary Figure 9. SOD2 modulates the response to 6-thio-dG.

(a) SOD2 was silenced using shRNA. Transduced WM3000 cells were treated with DMSO or 1μM of 6-thio-dG and cell death was assessed by Annexin V/PI straining. (b) Cells were transduced with a SOD2 lentiviral construct or empty vector control (pLX304). Transduced cells were treated with increasing doses (1, 2.5 and 5 μM) of 6-thio-dG for 10 days and cell death was assessed. Data represent average of three independent experiments +/- SEM. *p <0.05 in unpaired Student's t-test.

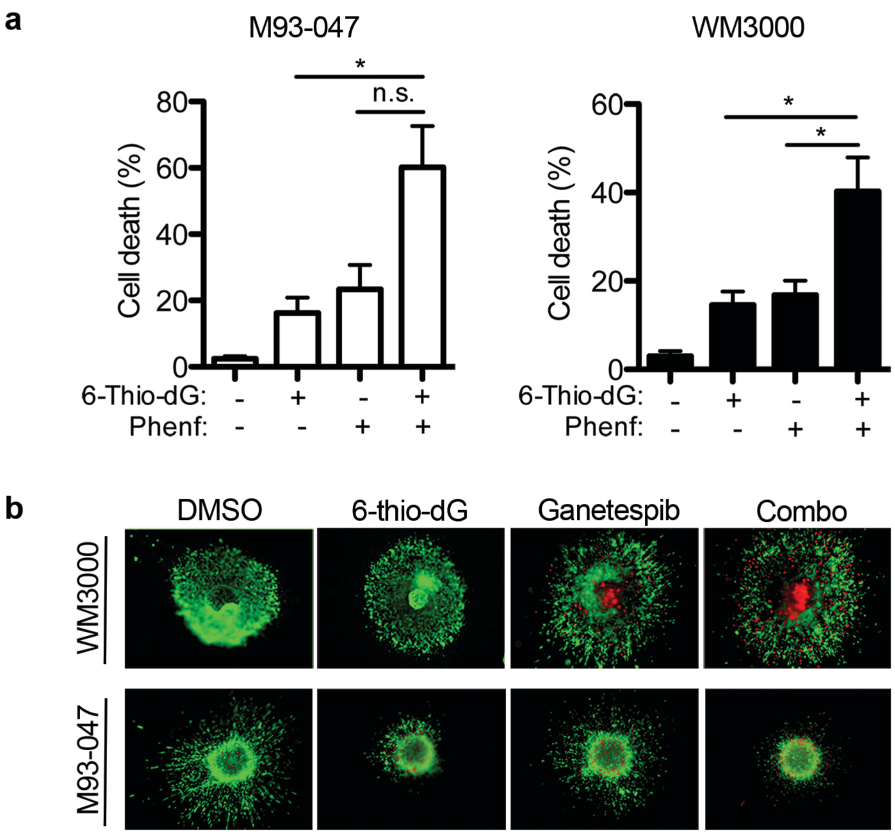

Supplementary Figure 10. Impairing mitochondrial function potentiates the effects of 6-thio-dG.

(a) $NRAS^{mut}$ melanoma cells were treated with 6-thio-dG (1µM) for 7 days total. At day 5, culture medium was replaced and cells were treated with 1 µM 6-thio-dG plus 1mM Phenformin for two more days. Percent cell death was determined by flow cytometry following staining of cells with Annexin V and Propidium iodide. Data represent average of three independent experiments +/- SEM. *p <0.05 in unpaired Student t test. (b) Collagen-embedded spheroids were treated with DMSO, 6-thio-dG (5µM) and Ganetespib (5µM) as in (a). On day 7, spheroids were stained with Calcein-AM (live cells; green) and EtBr (dead cells; red) and

imaged using an inverted microscope (4X). Representative merged images from three independent experiments are shown.
