# Supplementary material for: Exploiting TERT dependency as a therapeutic strategy for NRAS-mutant melanoma
Source: Oncogene. 2018 Apr 26;37(30):4058–72. doi: 10.1038/s41388-018-0247-7 (PMC6062502; doi:10.1038/s41388-018-0247-7)
Supplement: Supplementary file 2 — Supplemental material [file 41388_2018_247_MOESM2_ESM.docx]

**ONLINE SUPPLEMENTAL MATERIALS**

**EXPLOITING TERT DEPENDENCY AS A THERAPEUTIC**

**STRATEGY FOR NRAS MUTANT MELANOMA**

Patricia Reyes-Uribe, Maria Paz Adrianzen-Ruesta, Zhong Deng, Ileabett Echevarria-Vargas, Ilgen Mender, Steven Saheb, Qin Liu, Dario C. Altieri, Maureen E. Murphy, Jerry W. Shay, Paul M. Lieberman, and Jessie Villanueva.

Supplementary Tables 1-2

Supplementary Material and Methods

**Supplementary Tables**

**Supplementary Table 1*.* Genotype of NRAS mutant melanoma cell lines used in**

**the study**

| Cell line | NRAS mutation | TERT promoter  mutation |
| --- | --- | --- |
| WM3000 | Q61R | C/T (-124, -138, -139) |
| WM3451 | Q61K | T/C, C/T (-246, -139, -138) |
| M93-047 | Q61K | --- |
| WM852 | Q61R | C/C, C/T (-246, -139, -138) |
| WM1366 | Q61L | T/C, C/T (-138, -139) |
| WM3629 | G12D | T/C (-124) |
| WM4113 | Q61R | A/C (-57) |

| **Supplementary Table 2. Primers sequences** | | |
| --- | --- | --- |
| Gene | Forward Seq. 5’-3’ | Reverse Seq.5’-3’ |
| GAPDH | TGCCAATGATGACATCAAGAA | GGAGTGGGTGTCGCTGTTG |
| TERT | GCCGATTGTGAACATGGACTACG | GCTCGTAGTTGAGCACGCTGAA |
| PGC1 alpha | CTGCTAGCAAGTTTGCCTCA | AGTGGTGCAGTGACCAATCA |
| PGC1 beta | CAGACAGAACGCCAAGCATC | TCGCACTCCTCAATCTCACC |
| SOD2 | GGCCTACGTGAACAACCTGA | CCGTTAGGGCTGAGGTTTGT |
| GPX1 | TATCGAGAATGTGGCGTCCC | CAAACTGGTTGCACGGGAAG |
| UCP2 | CCTCTCCCAATGTTGCTCGT | GGCAAGGGAGGTCATCTGTC |

**Supplementary Material and Methods**

**Cell lines**

Human patient-derived melanoma cell lines were established in Dr. Meenhard Herlyn’s lab at The Wistar Institute following strict SOPs and quality controlled <https://www.wistar.org/sites/default/files/2017-11/Herlyn%20Lab%20-%20Cell%20Lines.xlsx>), except M93-047. NRAS mutant M93-047 (RRID:CVCL_G298) was derived from a metastatic axillary lymph node and was a generous gift of Dr. A. Weeraratna (The Wistar Institute). HCT116 and A549 cells were provided by M. Herlyn and S-K-NAS by C. Dang (The Wistar Institute). Primary human fibroblasts (FF2511) were obtained from neonatal foreskin samples from Lankenau Institute for Medical Research under approved IRB protocol # 21212264. Human skin samples were mechanically dissociated and fibroblasts cultivated in RPMI-1640 supplemented with 10%FBS. All cell lines were periodically authenticated by DNA finger printing using Life Technologies AmpFISTR Identifier microsatellite kit (Carlsbad, CA) and tested for mycoplasma by Lonza Mycoalert Assay (Basel, Switzerland). NRAS and BRAF-V600E mutation status was verified by direct Sanger sequencing.

**Reagents**

Small Molecule Inhibitors:

Trametinib (GSK1120212; S2673) and palbociclib (PD-0332991; S1116) were purchased from Selleckchem (Houston, TX).

Antibodies:

NRAS (SC-519l; Santa Cruz Biotechnology, Dallas, TX)

TERT (600-401-252; Rockland, Limerick, PA)

Cyclin A (NCL-Cyclin A; Leica Biosystems, Newcastle, UK)

53BP1 (IHC-00001; Bethyl Laboratories, Montgomery, TX)

gH2AX (05-636; Millipore, Billerica, Massachusetts, United States)

SOD2 (13141 Cell Signaling Technology [CST], Danvers, MA**)**

ERK (9107; CST)

pERK (4370; CST)

pRb-Ser807/811 (9308; CST)

Cleaved caspase-3 (9661; CST)

gH2AX [FACS] (9718; CST)

Vinculin (SAB4200080) and Actin antibodies (Sigma-Aldrich, St. Louis, MO) were used as loading controls.

**Immunoblotting**

For western blot analysis, cells were washed with cold PBS containing 100 µM Na_3_VO_4_, scraped, collected by centrifugation, and quick-frozen in dry ice before lysis. Cells were lysed with TNE buffer (50 mM Tris-HCl, 2 mM EDTA, 25 mM NaCl, 1% NP40 and protease inhibitors) and equal amounts of protein (30-50 µg) were subjected to SDS-PAGE and proteins transferred onto nitrocellulose membranes (Bio-Rad).

## **PCR Array**

For gene expression profiling using the human cellular senescence RT2 Profiler PCR array, 1µg of mRNA was used for cDNA synthesis with RT2 First Strand Kit (Applied Biosystems, Waltham, MA). cDNA and SYBR Green mastermix dispensed into PCR array plate and placed in the 7500 real-timer cycler (Applied Biosystems).

**Quantitative real time PCR (qRT-PCR)**

Total RNA (1 µg) was reverse transcribed using Maxima First-Strand cDNA synthesis kit (ThermoFisher Scientific). Fast SYBR Green Master Mix (Applied Biosystems) was used with 100 ng cDNA template and 250 nM primers for the evaluation of target gene expression. Primers sequences are listed in Table S2. Amplification was performed in triplicate using ABI PRISM 7500 Fast RT PCR System (Applied Biosystems) and expression of mRNA was assessed using the ∂Ct method.

**TIF analysis**

For TIF analysis, indirect immunofluorescence (IF) combined with fluorescence in situ hybridization (FISH) was performed as previously described with some modifications ^(56)^. Briefly, 1 x 10^5^ cells grown on coverslips were fixed for 15 min in 1% paraformaldehyde in PBS at room temperature, followed by permeabilization for 15 min in 1x PBS/0.3% Triton X-100 at RT. After, cells were incubated for 60 min in PBG blocking solution (0.5% BSA, 0.2% fish gelatin in 1x PBS) before immuno-staining. Primary antibodies were prepared in blocking solution at the following dilutions: anti-53BP1 (1:500; Bethyl Laboratories), and anti- γH2AX (1:500; Millipore). After IF, cells were fixed in 4% paraformaldehyde in 1X PBS for 10 min and dehydrated in ethanol series (70%, 95%, 100%). Coverslips were incubated 3 min at 80-85°C in hybridization mix [70% formamide, 10mM Tris-HCl, pH 7.2, and 0.5% blocking solution (Roche)] containing telomeric PNA-Tamra-(CCCTAA)3 probe, followed by hybridization at room temperature for 2 hrs. Coverslips were washed twice for 15 min each with 70% formamide, 10 mM Tris-HCl (pH 7.2), and 0.1% BSA, followed by three 5 min washes with 0.15 M NaCl, 0.1 M Tris-HCl (pH 7.2), and 0.08% Tween-20. Nuclei were counterstained with 0.1 [µg/ml] DAPI in blocking solution and slides were mounted with Fluoromount-G (0100-01, SouthernBiotech). Images were captured with a 100x objective on a Nikon E600 Upright microscope (Nikon Instruments, Inc., Melville, NY) using ImagePro Plus software (Media Cybernetics, Silver Spring, MD) for image processing. Cells with > 4 53BP1 or γH2AX foci co-localizing with telomere DNA foci were scored as TIF positive.
